# Supplementary material for: Using Polygenic Scores for Circadian Rhythms to Predict Wellbeing, Depressive Symptoms, Chronotype, and Health
Source: J Biol Rhythms. 2024 Mar 1;39(3):270–81. doi: 10.1177/07487304241230577 (PMC11141090; doi:10.1177/07487304241230577)
Supplement: sj-docx-1-jbr-10.1177_07487304241230577 – Supplemental material for Using Polygenic Scores for Circadian Rhythms to Predict Wellbeing, Depressive Symptoms, Chronotype, and Health [file sj-docx-1-jbr-10.1177_07487304241230577.docx]

**Using Polygenic Scores for Circadian Rhythm to predict**

**Wellbeing, Depressive Symptoms, Chronotype, and Health**

Landvreugd, A., Pool, R., Nivard, M., & Bartels, M.

## Appendix

# 1. Methods for generating polygenic scores in NTR

## Summary statistics

The polygenic scores (PGSs) in the Netherlands Twin Register (NTR) sample were based on the discovery GWAMA of Ref. Jones et al., 2019 for phenotype Morningness, and GWA of Ref. Ferguson et al. 2019 for phenotype Relative Amplitude. We retained variants for which the effect allele frequency (EAF) was 0.01 ≤ EAF ≤ 0.99. Variant EAF and effect sizes were aligned with the NTR reference for the 1000 genomes variants. Discovery variants that were not part of this reference were discarded.

## Generation of weighted effect sizes

### LDpred v0.9

The processed summary statistics were taken as input for the LDpred 0.9 software (Vilhjálmsson et al., 2015). For estimating the target LD structure we (1) used a selection of unrelated individuals in the NTR sample and (2) selected a set of well-imputed variants in the NTR sample. The parameter ld_radius was set by dividing the number of variants in common (from the output of the coordination step) by 12000. Note that for the coordination step we provided the median sample size as input value for N. For the LDpred step we applied the following thresholds for fraction of variants with non-zero effect (in addition to the default infinitesimal model): --PS=0.5,0.3,0.2,0.1,0.05,0.01.

### SBayesR

The SBayesR method is implemented in the gctb software (Lloyd-Jones et al., 2019; Zeng et al., 2018). For computing the weighted effect sizes we took the above processed summary statistics as input and applied the following arguments:
--sbayes R; --pi 0.95,0.02,0.02,0.01; --gamma 0.0,0.01,0.1,1; --chain-length 40000; --burn-in 4000; --out-freq 10. Note that for practical and computaional reasons, we applied the LD matrix as provided by the authors of Lloyd-Jones et al., 2019, which is based on 2.8 million common variants in a random selection of 50000 individuals of the UK biobank sample (Lloyd-Jones., 2019).

## Scoring weighted effect sizes in the NTR sample

We used the plink2 software package (Chang et al., 2015, Plink 2.0) for generating the PGSs by applying the --score option to the input weighted effect sizes and the genotype data set. As scoring genotype data sets, we use the entire NTR sample or a subset of (non)transmitted paternal or maternal alleles. The latter subsets were generated by taking data from trios in the NTR genotype data set.

### LDpred v0.9

Of each set of weighted effect sizes (infinitesimal model and additional threshold, see above) we calculated the NTR PGSs over all genotype data sets. We applied the following thresholds for fraction of variants with non-zero effect (in addition to the default infinitesimal model): --PS=0.01,0.05,0.1,0.2,0.5.We utilized the scores of the entire NTR sample.

### SBayesR

For the set of weighted effect sizes taken from gctb --sbayes R, we calculated the NTR PGSs over all genotype data sets. We utilized the scores of the entire NTR sample.

## References

Chang, Christopher C., Carson C. Chow, Laurent Cam Tellier, Shashaank Vattikuti, Shaun M. Purcell, and James J. Lee. ‘Second-Generation PLINK: Rising to the Challenge of Larger and Richer Datasets’. *GigaScience* 4 (2015): 7. <https://doi.org/10.1186/s13742-015-0047-8>.

Ferguson, Amy, Laura M. Lyall, Joey Ward, Rona J. Strawbridge, Breda Cullen, Nicholas Graham, Claire L. Niedzwiedz, et al. ‘Genome-Wide Association Study of Circadian Rhythmicity in 71,500 UK Biobank Participants and Polygenic Association with Mood Instability’. *EBioMedicine* 35 (September 2018): 279–87. <https://doi.org/10.1016/J.EBIOM.2018.08.004>.

Jones, Samuel E., Jacqueline M. Lane, Andrew R. Wood, Vincent T. van Hees, Jessica Tyrrell, Robin N. Beaumont, Aaron R. Jeffries, et al. ‘Genome-Wide Association Analyses of Chronotype in 697,828 Individuals Provides Insights into Circadian Rhythms’. *Nature Communications 2019 10:1* 10, no. 1 (January 2019): 1–11. <https://doi.org/10.1038/s41467-018-08259-7>.

GCTB sparse shrunk LD matrices from 2.8M common variants from the UK

Lloyd-Jones, L. R., Zeng, J., Sidorenko, J., Yengo, L., Moser, G., Kemper, K. E., Wang, H., Zheng, Z., Magi, R., Esko, T., Metspalu, A., Wray, N. R., Goddard, M. E., Yang, J., & Visscher, P. M. (2019). Improved polygenic prediction by Bayesian multiple regression on summary statistics. *Nature Communications*, *10*(1), 5086. <https://doi.org/10.1038/s41467-019-12653-0>

Lloyd-Jones, L., Zeng, J., Sidorenko, J., Yengo, L., Moser, G., Wang, H., Zheng, Z., Magi, R., Esko, T., Wray, N., Goddard, M., Yang, J., & Visscher, P. (2019). Improved polygenic prediction by Bayesian multiple regression on summary statistics. *Nature Communications*, *10*, 5086. <https://doi.org/10.1038/s41467-019-12653-0>

PLINK 2.0. https://www.cog-genomics.org/plink/2.0/.

Vilhjálmsson, B. J., Yang, J., Finucane, H. K., Gusev, A., Lindström, S., Ripke, S., Genovese, G., Loh, P.-R., Bhatia, G., Do, R., Hayeck, T., Won, H.-H., Schizophrenia Working Group of the Psychiatric Genomics Consortium, Discovery, Biology, and Risk of Inherited Variants in Breast Cancer (DRIVE) study, Kathiresan, S., Pato, M., Pato, C., Tamimi, R., Stahl, E., Zaitlen, N., … Price, A. L. (2015). Modeling Linkage Disequilibrium Increases Accuracy of Polygenic Risk Scores. *American Journal of Human Genetics*, *97*(4), 576–592. <https://doi.org/10.1016/j.ajhg.2015.09.001>

Zeng, J., de Vlaming, R., Wu, Y., Robinson, M. R., Lloyd-Jones, L. R., Yengo, L., Yap, C. X., Xue, A., Sidorenko, J., McRae, A. F., Powell, J. E., Montgomery, G. W., Metspalu, A., Esko, T., Gibson, G., Wray, N. R., Visscher, P. M., & Yang, J. (2018). Signatures of negative selection in the genetic architecture of human complex traits. *Nature Genetics*, *50*(5), 746–753. <https://doi.org/10.1038/s41588-018-0101-4>

**2. Formula for the expected effect size**

Although the PRS effect size of R^2^ = 1.5% seems small (implying a correlation of *r* = 0.12), it is in line with the expectations for a highly polygenic trait (Boyle et al., 2017). The expectation for the R^2^ can be expressed as a function of 1) the SNP-heritability, which is ~12% for Morningness, 2) the sample size (N), which is 499k for the GWAS discovery sample we had access to (Jones et al., 2019), and 3) the number of causal loci (M), which is unknown but in the analysis of other polygenic traits 125.000 has been used.

The formula is (Pasaniuc & Price, 2017):

$$R^{2}= \frac{{h^{2}}_{snp}}{1 + \frac{M}{N* {h^{2}}_{snp}}}$$

As M is constant, as N goes up, the term $\frac{M}{N* {h^{2}}_{snp}}$ slowly shrinks, and eventually the R^2^ is asymptotically approaches ${h^{2}}_{snp}$, which has been observed for standing height at a GWAS sample size of ~ 6.000.000.

For Morningness, the equation suggests:

$$R^{2}= \frac{0.12}{1 + \frac{125.000}{449.000* 0.12}} 0.036$$

Subtle uncertainties in the various parameter’s mater greatly. For example, the estimated SNP-heritability for Morningness is ~ 12% resulting in an expected R^2^ = 3.6%, while a SNP-heritability of 11% or 10.5% would result in R^2^ = 3.1% or 2.8%.

## References

Boyle, Evan A., Yang I. Li, and Jonathan K. Pritchard. ‘An Expanded View of Complex Traits: From Polygenic to Omnigenic’. *Cell* 169, no. 7 (15 June 2017): 1177–86. <https://doi.org/10.1016/j.cell.2017.05.038>

Pasaniuc, Bogdan, and Alkes L. Price. ‘Dissecting the Genetics of Complex Traits Using Summary Association Statistics’. *Nature Reviews. Genetics* 18, no. 2 (February 2017): 117–27. <https://doi.org/10.1038/nrg.2016.142>.
